# Real-world energy data of 200 feeders from low-voltage grids with metadata in Germany over two years

**DOI:** 10.1038/s41597-026-08152-9

**Published:** 2026-09-05

**Authors:** Manuel Treutlein, Pascal Bothe, Marc Schmidt, Roman Hahn, Oliver Neumann, Frederik Gielnik, Ralf Mikut, Veit Hagenmeyer

**Affiliations:** 1 Institute for Automation and Applied Informatics (IAI), Karlsruhe Institute of Technology (KIT), Karlsruhe, Germany; 2 AI & Analytics, Netze BW GmbH, Stuttgart, Germany; 3 Institute for Electric Energy Systems and High-Voltage Technology (IEH), Karlsruhe Institute of Technology (KIT), Karlsruhe, Germany

## Abstract

The last mile of the distribution grid is crucial for a successful energy transition, as more low-carbon technology like photovoltaic systems, heat pumps, and electric vehicle chargers connect to the low-voltage grid. Despite considerable challenges in operation and planning, researchers often lack access to suitable low-voltage grid data. To address this, we present the FeederBW dataset with data recorded by the German distribution system operator Netze BW. It offers real-world energy data from 200 low-voltage feeders over two years (2023-2025) with weather information and detailed metadata, including changes in low-carbon technology installations. The dataset includes feeder-specific details such as the number of housing units, installed power of low-carbon technology, and aggregated industrial energy data. Furthermore, high photovoltaic feed-in and one-minute temporal resolution makes the dataset unique. FeederBW supports various applications, including machine learning for load forecasting, conducting non-intrusive load monitoring, generating synthetic data, and analyzing the interplay between weather, feeder measurements, and metadata. The dataset reveals insightful patterns and clearly reflects the growing impact of low-carbon technology on low-voltage grids.

## Background & Summary

The low-voltage (LV) grid is pivotal in facilitating the energy transition towards renewable energy sources and increased electrification. Integrating essential low-carbon technology (LCT) such as heat pumps, electric vehicles (EVs), and photovoltaic (PV) systems into the LV grid in substantial numbers has become an indispensable task. This shift requires significant modifications in the operation and planning of LV grids, which were traditionally designed for fossil-fuel-based systems. For example, planners and operators have to deal with much more data from decentralized generation and consumption^1^. Consequently, a large and active research community has been focusing on developing innovative approaches to make this transition economically viable. A critical component of this research is the availability of realistic data, a need that is further amplified by advancements in machine learning and artificial intelligence which require large amounts of data^2^.

The energy transition in the LV grid presents diverse challenges from the perspective of distribution system operators (DSOs). Grid planners are tasked with dimensioning existing and new LV grids to ensure adequate capacity. To achieve this, they employ power flow (PF) calculations, coincidence factors, or load and generation profiles. However, the effectiveness of these methods hinges on the availability of realistic data, which should ideally include power measurements of LCT for which high future penetration rates are anticipated. Regarding the operation of LV grids, it is increasingly critical for operators to have grid state estimations (SEs) based on real measurements and models. This enables continuous monitoring but also serves as a prerequisite for controlling load and generation to prevent overloads or voltage band violations in cables, overhead lines, or transformers.

Furthermore, real measurement data is essential for training machine learning models that support DSO processes. Nevertheless, many LV grids lack sufficient measurement devices, because they are not yet installed or are too expensive. In addition, future measurements are inherently unavailable. Consequently, models based on real-time data and realistic historical data are crucial. For instance, these models can produce load forecasts for LV feeders for the following day or help addressing the gaps left by insufficient measurement infrastructure.

Several review papers focusing on LV grids and smart grids, which include overviews of public datasets, have been published in recent years^2–4^. However, authors across these review papers consistently highlight the lack of sufficient public datasets. Research on non-public datasets is frequently observed in LV load forecasting^2^, leading to non-reproducible research and contradicting good scientific practice. A major issue is that many power companies refuse to provide public datasets due to privacy or marketing concerns^4^.

To identify the dataset gap filled by the *FeederBW* dataset, we provide an overview of existing public LV grid datasets. We exclude datasets with highly aggregated load or market data at higher system levels, such as those presented in^4^. Furthermore, we distinguish for public LV grid datasets between (1) residential, (2) commercial/industrial, and (3) grid-measured datasets^2^.

The majority of these datasets consist of smart meter data collected from residential buildings (1). These datasets provide real-world load data from single households^5,6^ and are often enriched with metadata, such as information about the appliances present in the respective buildings^7^.

In contrast, commercial and industrial datasets (2) include commercial buildings in addition to residential buildings^8,9^ or focus exclusively on industrial facilities and their associated metadata^10^. These datasets are valuable for analyzing energy consumption patterns in non-residential settings.

Residential, commercial, and industrial datasets can include data about the grid, such as grid topology^9^. However, these datasets typically do not incorporate measurements of DSO equipment in the LV grid. In the *FeederBW* dataset, we address this gap by focusing on measurements of the LV feeders. This is particularly significant given the scarcity of public datasets that include such measurements, making LV feeder data strongly underrepresented.

Datasets with measurements in the LV grid (3) are scarcely published. Often, available grid datasets are aggregations of smart meter values from households. While smart meter data from individual customers can be aggregated to the LV feeder level, it is important to note that this data is not directly measured at the LV feeder and is therefore not considered as LV grid-measured dataset in this paper. For example, such datasets typically do not include phase unbalances or a mix of residential and commercial customers. The approach of aggregating data from various consumers and producers at the smart meter level is exemplified in benchmark datasets such as SimBench^11^. Similarly, projects and datasets like the data from Hvaler in Norway^12^, Weave Smart Meter Data^13^, the Iowa Distribution test system project, the SSEN Distribution LV Feeder Usage data, and UK Power Networks data provide smart meter consumption data aggregated at the LV feeder level^2^.

Unlike grid-measured datasets, grid datasets often include a grid topology, which is essential for applications such as PF analysis, SE, and other grid-related studies^3^. Existing LV grid datasets serve various purposes, for example providing benchmark grids and test systems like the IEEE and CIGRE test cases^14^. However, these datasets frequently lack time-series load data or are limited to specific grid configurations^15^. As a result, researchers often rely on semi-synthetic data or other real-world load time-series for grid calculations.

In contrast, the *FeederBW* dataset provides 200 LV feeders with one-minute temporal resolution, offering a large volume of load and generation data over two years. This makes it particularly valuable for machine learning (ML) applications. We include recommended train-test splits for ML experiments in the *Usage Notes* at the end of the paper. While the *FeederBW* dataset does not include a grid topology, as it is not designed for grid calculations such as solving PFs or SEs, it can supplement such calculations by providing load and generation patterns from real-world time-series data.

Table 1 provides an overview of LV grid datasets that are closely related to our dataset. We have included datasets that contain time-series power or load measurements, either from medium-voltage (MV) to LV substations or from LV feeders. The small number of five datasets already underlines the necessity for more published grid-measured datasets.

**Table 1:** Literature and datasets of LV grid-measured datasets with real measurement time-series of power or load for MV/LV substations (sub.), LV feeders or cable distribution cabinets.

Name	Country^a^	Period (Years)	Equipment of DSO	Resolution	Measurements^b^	Weather data^c^	Area type (Location)
BigDEAL^17^	US	2015–2018 (4y)	3 sub.	1h	load	T	unclear (USA)
OPSCI TP Gradisce^18^	SI	2019–2023 (4y)	1 sub.	15 min	P, Q	not given	unclear (Slovenia)
NTVV^19^	UK	2014–2017 (4y)	316 sub. including feeders	5s	V, I, P, Q, VHC	not given	suburban (Bracknell)
Flexible Networks for a Low Carbon Future^20^	UK	2012–2014^d^ (1y)	184 sub.	30 min	unclear	not given	unclear (St Andrews, Wrexham, Whitchurch)
Rolle Hierarchical Benchmark^21^	CH	2018 (1y)	24 sub. & cabinets	10 min, (weather: 1h, 12h)	for each phase: P, Q, V, THD, *ω*	T, GHI, GNI, RH, Pr, *W*_*S*_, *W*_*dir*_	rural (Rolle)
**FeederBW** [this paper]	DE	2023–2025 (2y)	200 feeders	1 min (weather: 1h)	for each phase: I, PF; V, P, Q	DirR, DifR, T, Prec, SH, Hum, WG, MW, ZW	mainly rural (Baden-Württemberg)

^a^country codes: US (United States of America), UK (United Kingdom), SI (Slovenia), CH (Switzerland), DE (Germany). ^b^abbreviations measurements: V (voltage), I (current), P (active power), Q (reactive power), VHC (voltage harmonic content), THD (total harmonic distortion), *ω* (voltage frequency), PF (power factor). ^c^abbreviations weather: T (Temperature), GHI (global horizontal irradiance), GNI (global normal irradiance), RH (relative humidity), Pr (pressure), *W*_*S*_ (wind speed), *W*_*dir*_ (wind direction), DirR (direct radiation), DifR (diffuse radiation), Prec (precipitation), SH (snow height), Hum (humidity), WG (max wind gust), MW (meridional wind), ZW (zonal wind). ^d^project duration of three years, duration of measurements is one year according to^2^.

The available datasets in Table 1 have several limitations that the FeederBW dataset addresses. There is a lack of LV feeder data specific to Germany, a gap filled by FeederBW with data from Baden-Württemberg. In comparison to other countries, the power at the LV feeder is characterized by a high degree of PV generation^16^. Temporally, FeederBW provides more recent data from 2023 to 2025, aligning with the 1-4 year scope of the other datasets (2012–2023). Existing datasets often include only a few substations or LV feeders, limiting their use for ML^17,18^. FeederBW offers data from 200 LV feeders over two years, including the diversity of LV feeders for a complete region and supporting robust training and evaluation of models.

Minute-level resolution is rare in Table 1, with only^19^ providing a higher resolution of 5 seconds. FeederBW provides with minute-level resolution the second highest resolution. Additionally, while many datasets focus solely on load measurements^17^, FeederBW includes comprehensive data including currents, power, power factor and voltage. Lastly, weather data is often lacking^18–20^ or scarce^17^ in existing datasets. FeederBW includes extensive local weather data, crucial for accurate predictions given the increasing dependency of load and generation on weather conditions.

Most notably, *FeederBW* includes extensive metadata about customers and their devices, such as heat pumps, electric vehicles or the number of housing units in the LV grid. Such details are largely absent in other datasets, limiting the interpretability. While some datasets in Table 1 provide measurements from the underlying or overlying grid^19,21^ or include grid topology information^18^, none match the depth of consumer and producer details offered by *FeederBW*. Additionally, *FeederBW* addresses the lack of representation of PV generation in existing datasets. Many datasets primarily focus on load data, despite the growing dominance of PV in LV grids. *FeederBW* includes numerous feeders with significant PV feed-in, providing a more comprehensive view of modern energy systems. Lastly, *FeederBW* explicitly includes measurement data from LV feeders characterized by industry and commerce, a feature often missing or unclear in other datasets.

The FeederBW dataset supports a variety of applications and research in energy systems. It can be used to develop and evaluate diverse ML models, including load forecasting^2,3^, building load and generation profiles, and non-intrusive load monitoring (NILM) at the LV feeder level. For example, a similar dataset was used in^22^ to train a model for pseudo-measurements. The model uses feeder metadata, weather data, calendar data and the timestamp encoding to estimate the load and generation of non-measured LV feeders. The active power measurements are used as target variable.

Additionally, the dataset enables the creation of synthetic data for grid studies, allowing for more realistic simulations. For this purpose, the feeder metadata helps to make informed decisions. Furthermore, the feeder metadata is a good indicator of concept drift. Hence, the dataset is valuable for developing and evaluating concept drift detection algorithms. Researchers can also work on data improvement techniques, such as data imputation, anomaly detection, and identifying and correcting metadata errors. Furthermore, the dataset facilitates analysis and understanding of grid dynamics, including the coincidence of devices/households at the LV feeder level, weather influences on load and generation, and relationships between power factors, active and reactive power, and phase currents. The measurement methodology is well suited for DSO tasks such as capacity planning of LV feeders or detecting asset overloading during operation. However, since the rated current is not given per LV feeder in the dataset, it is not possible assessing the proximity of feeders to thermal limits. Furthermore, advanced power quality analysis such as analyzing voltage and current distortions or voltage unbalance are out of scope for the recorded dataset as they require a more precise methodology for measuring.

Many existing datasets, as shown in Table 1, suffer from poor curation, including missing background information, inaccessibility (e.g.,^18^, access failed on 24 September 2025), and lack of identifiers like digital object identifiers (DOIs). To ensure the quality and usability of our datasets, we adhere to the *FAIR principles*^23^. We use Zenodo, which assigns DOIs to each entry, to enable findability. Zenodo also provides multiple retrieval methods and ensures long-term accessibility. The download of our dataset is manageable on conventional computers. We employ standard data types and file formats for interoperability and provide extensive metadata and licensing information to clarify reusability and provenance.

## Methods

**Fig. 1:**
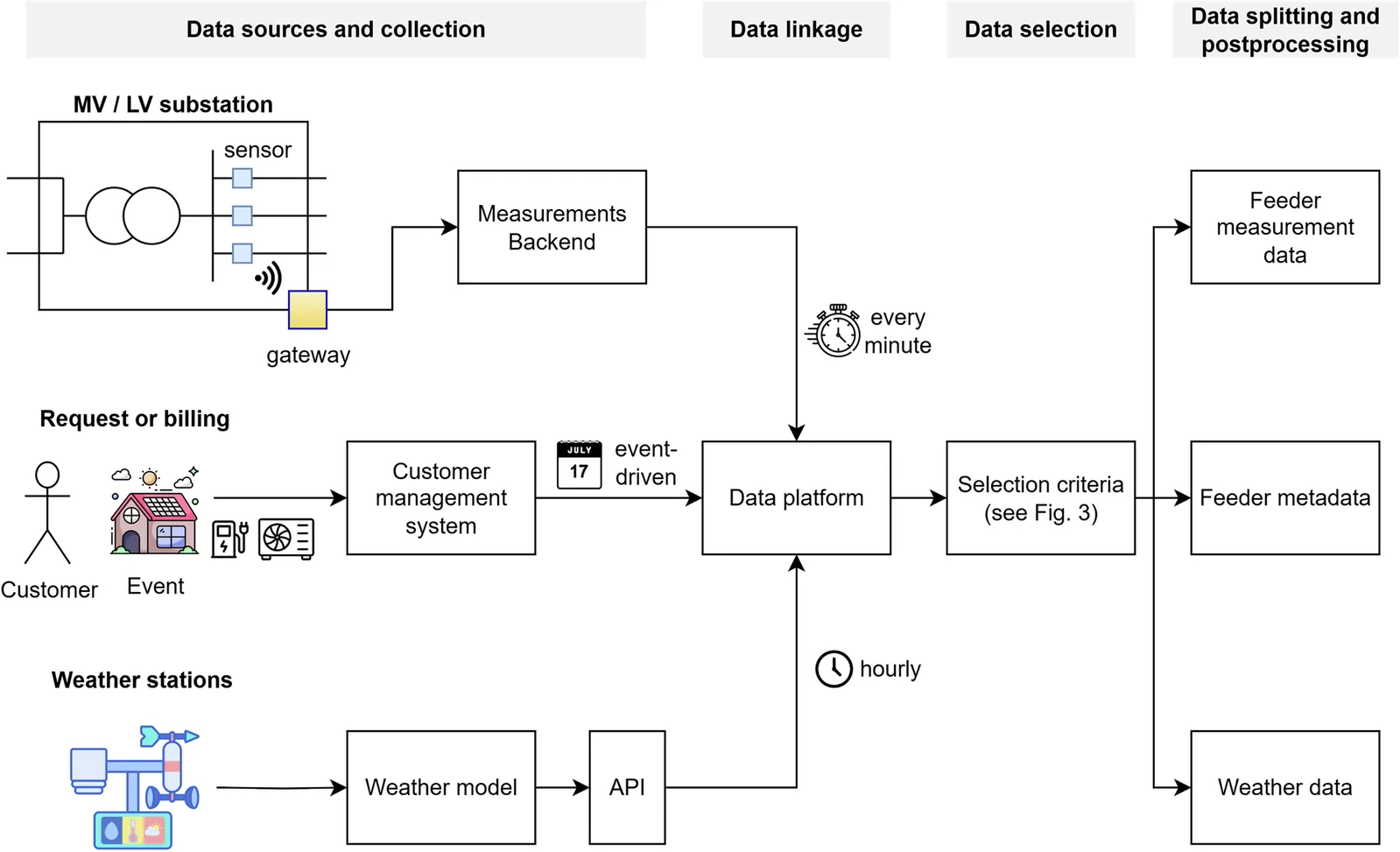
The data collection process of the feeder, meta and weather data. The collected data is combined in a data platform, the data is selected and again split into three data records. [icons in image: flaticon.com].

The published data is real-world data collected and used at Netze BW, the largest DSO in the federal state of Baden-Württemberg in the southwest of Germany. We describe the collection of the feeder data which is enriched by metadata and weather data on a data platform. Furthermore, we describe which criteria were applied to derive the final 200 LV feeders. The data process is shown in Fig. 1.

### Data sources and collection

#### Feeder measurement data

**Fig. 2:**
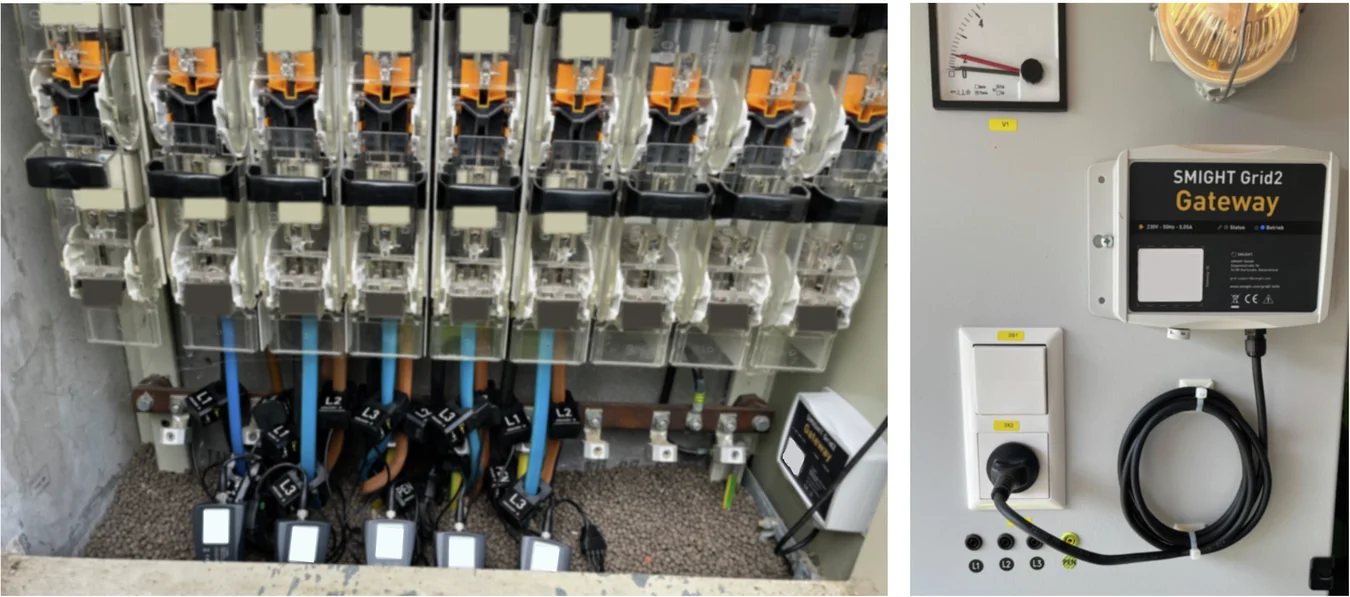
Example for sensors and gateway used in the MV/LV substations to measure the LV feeders. [image: Netze BW GmbH].

The measurement of the feeders is conducted using *SMIGHT Grid 2 (gateway + sensor)*^24^ from the company SMIGHT, installed in MV/LV substations of the DSO Netze BW in the southwest of Germany (see Fig. 2). Hence, the measurement data is provided by Netze BW and represents an excerpt from the regular grid monitoring data^25^. The authorization to share the data was obtained through an internal process at Netze BW.

The sensors are clipped around each phase of a LV feeder cable. The transformer is not directly measured. The sensors capture the root mean square (RMS) current for each phase, including protective earth neutral (PEN) for each minute. Power supply for the sensors is achieved through energy harvesting, eliminating the need for an external power source. However, this necessitates a minimum current for the sum of all phases plus PEN of 32 A as energy supply for a stable operation of the sensor. The measurement accuracy is 3% between 5–400 A.

The collected data is transmitted to a gateway. It measures the busbar voltage synchronized to the current measurement by directly capturing the voltage of its own energy supply. Since the energy supply of the gateway is provided by only one phase, we only measure the voltage on one phase. The measurement range is between 85–264 V with 3% accuracy. Phase angle, the direction of the current flow and the distribution between active and reactive part of the current are determined based on the phase shift between current and voltage. Finally, the gateway forwards the data to the backend. It is important to note that there is only one voltage measurement for the entire substation but not for individual feeders or phases. Consequently, feeders from the same substation share identical voltage measurements.

#### Feeder metadata

The feeder metadata originates from the customer management system at Netze BW and is provided by the DSO^25^. Similar to the feeder measurement data, the authorization to share the data was obtained through an internal process at Netze BW. It can be categorized into three types: the number of connected objects, the installed power of consumers or producers, and the average hourly energy consumption of industry and commerce customers. Note that the industry values are averages over several months or years and not hourly data. The data collection process spanned several decades and involved numerous employees across various regions, leading to slight variations in the methodology in addition to changes in the data acquisition process over time. Unlike feeder measurement data and weather data, this dataset is characterized by event-driven entries, triggered by customer connection requests such as new PV installations. Furthermore, we update the entry when new average energy consumption values for industry and commerce are recorded. Finally, the metadata of a LV feeder given in this dataset describes the sum of all registered connected objects, the sum of installed power for consumers and producers and the current average energy consumption for industry and commerce.

#### Weather data

The weather data is retrieved from the SANDY Weather API of the Datalab (previously SANDY Energized Analytics) of the company EnBW^26^. Netze BW is a subsidiary of EnBW and the Datalab of EnBW provides the weather data for this publication upon internal request. The SANDY Weather API provides hourly weather data based on the postal code where the substation of the LV feeder is located. The weather data provided by the API originates from the numerical weather prediction (NWP) model ICON-D2 from the German Meteorological Service (DWD)^27^. The model relies on data from several hundred weather stations across Germany and offers spatially resolved weather forecasts at a 2 km grid resolution. The statistical processing of a meteorological variable by the DWD is dependent on the type, such as averaging for radiation over the hour or accumulating for precipitation within an hour, and is included in the table describing the data record in the next section. The forecasts are provided every three hours by the DWD. The SANDY Weather API is caching the forecasts and updates existing forecasts with the most recent predictions. The weather data in the published dataset is retrieved from the SANDY Weather API for historical points in time, hence all values in the weather data represent very short-term forecasts with a forecast horizon of one to three hours. To provide data at the postal code level, all grid points of a meteorological variable within the postal code area are aggregated. Aggregation is done by the mean value, except for the maximum wind speed.

### Data linkage

The linkage of the data was conducted on a data platform introduced in^28^. We combined customer data, such as the number of installed PV systems or EV chargers, with measurements based on the grid topology to obtain the feeder metadata. In the subsequent step we retained only non-meshed grids to ensure that the feeder metadata could be clearly assigned to a single LV feeder rather than multiple feeders. The LV feeder is situated in an area identified by a postal code, which was used to match weather data with feeder measurement data and feeder metadata.

### Data selection process

**Fig. 3:**
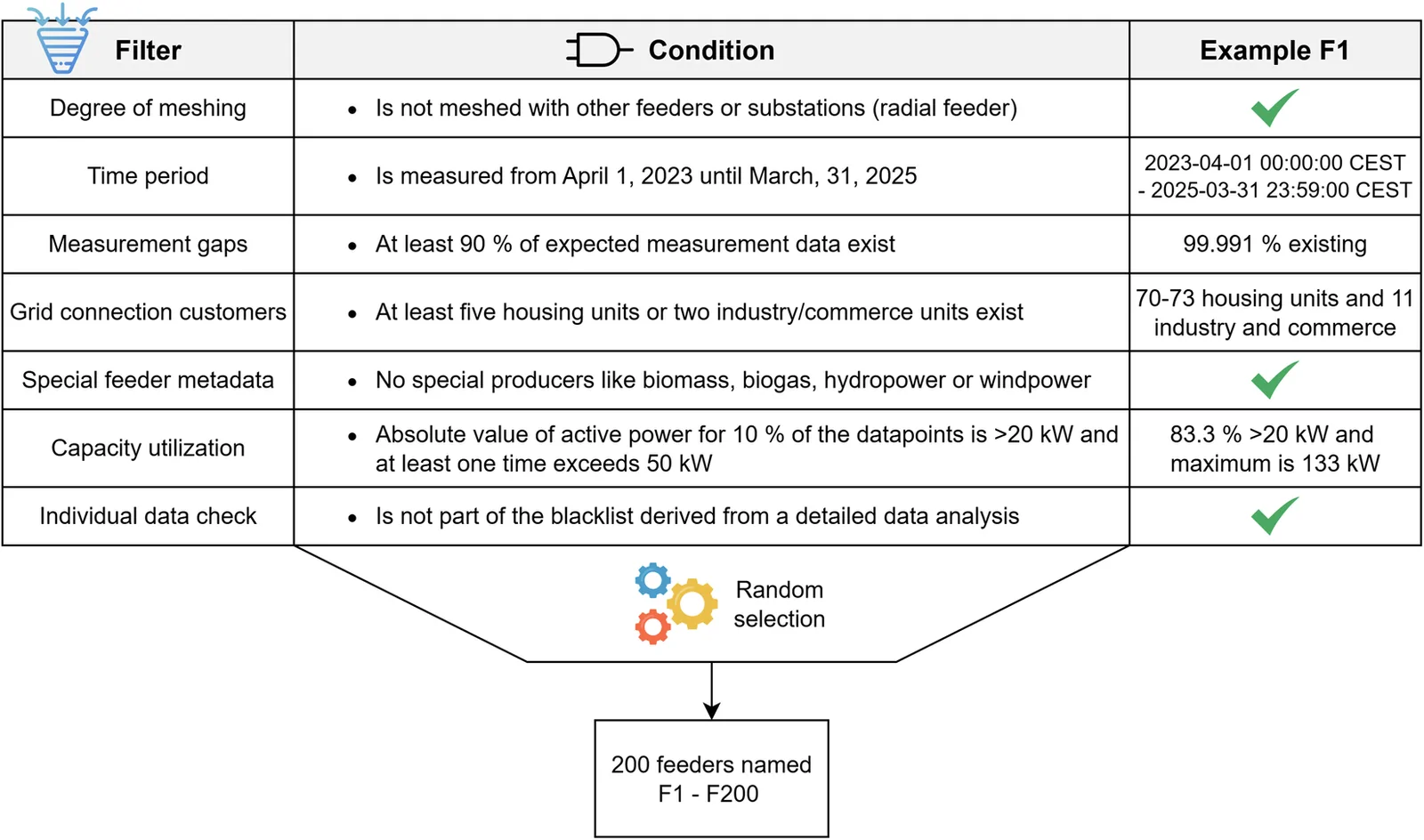
Selection criteria for the 200 LV feeders published in the FeederBW dataset, exemplified for feeder *F*_1_. All the published feeders fulfill all conditions. Refer to Figure 1 for the complete data process where the data selection is embedded. [icons in image: flaticon.com].

Every LV feeder included in the *FeederBW* dataset must satisfy all conditions outlined in Fig. 3.

#### Degree of meshing

This filter ensures the feeder is radial, preventing interconnections with other feeders or substations. Thereby we ensure that the load and feed-in of producers and consumers given in the feeder metadata can be fully assigned to the feeder without splitting the power flow across multiple feeders.

#### Time period

This filter ensures measurements span from April 1, 2023, to March 31, 2025, providing a two-year window which allows meaningful train-test splits for ML applications (see *Usage Notes*).

#### Measurement gaps

This filter guarantees that at least 90% of expected measurement data is available, reducing errors and minimizing preprocessing for users.

#### Grid connection customers

This filter requires at least 5 housing units or 2 industry/commerce units to ensure data privacy, complementing the pseudonymization of feeder names.

#### Special feeder metadata

This filter excludes feeders with rare producers like biomass, biogas, hydropower, or wind power due to their unique and sparse data.

#### Capacity utilization

This filter ensures that the absolute value of the active power exceeds 20 kW for 10% of data points and reaches at least 50 kW once, focusing on feeders with significant capacity utilization, which are the crucial LV feeders for DSOs with respect to operation and planning.

#### Individual data check

This filter uses a blacklist based on detailed analysis to exclude feeders with uncorrectable data quality issues, such as topology changes.

Finally, 200 feeders are randomly selected for publication. While these criteria introduce bias, they ensure high data quality, privacy, information security, and relevance for LV feeders with notable capacity utilization. As an example, Fig. 3 shows that the criteria are fulfilled for feeder *F*_1_.

### Data splitting and postprocessing

For the data processing steps, such as data selection, it was convenient to have the data combined to apply filters. For publication, the data is split into feeder measurement data, feeder metadata, and weather data to reduce storage requirements due to different temporal resolutions. The resolution of the feeder measurement data is one minute, whereas the resolution of the weather data is one hour. The feeder metadata can be considered as event data, remaining constant until a new consumer or producer is registered or deregistered, or the average hourly energy consumption of industry/commerce changes.

For 51 LV feeders, all PEN values are in the range 1–2 A. The same accounts for the LV feeder *F*_181_ in the first months. The values for these feeders are replaced with Null, because it can be assumed that the instrument transformer could not be installed properly around the PEN cable. Further measurement data or weather data was not corrected.

## Data Records

The dataset is hosted on Zenodo^29^. As illustrated in Fig. 1, it is divided into three distinct data records: one for feeder measurement data, one for feeder metadata, and one for weather data. These records are summarized in Table 2 and the columns are presented in detail in Table 3 (feeder measurement data), Table 4 (feeder metadata) and Table 5 (weather data).

**Table 2:** Overview of the three published data records. Details about the columns of each data record are given in Table 3 (feeder measurement data), Table 4 (feeder metadata) and Table 5 (weather data).

Dataset	Feeder measurement data	Feeder metadata	Weather data
Number of columns	20	23	12
Number of rows	209,189,649	1,608	3,508,800
Temporal resolution	1 minute	event-driven	1 hour
File format	parquet	CSV	parquet
File size	8.5 GB (compressed)	178 KB	106 MB
Description	Voltage, power, currents, power factor of the LV feeder	Number of customer connection objects, installed power of consumers and producers and average consumption of industry/commerce at LV feeder	Weather data such as radiation, temperature and wind in postal code area of LV feeder

**Table 3:** Description for columns of the data record feeder measurement data. The data is recorded at a resolution of one minute, and each row is uniquely identified by the feeder and the timestamp (UTC). It contains voltage, power, currents for each phase including PEN and the power factor for each phase.

Column name	Unit	Data type	Description
feeder	count	int	Pseudonym of the feeder
timestamp_UTC	—	string	Timestamp in time zone UTC
timestamp_CET_CEST	—	string	Timestamp in local time zone of the DSO (Central European (Summer) Time)
voltage_one_phase_V (abbr. *U*)	V	float	Voltage of one phase measured at busbar/gateway
active_power_kW	kW	float	Calculated based on the voltage *U* of one phase and the respective active currents with $U\cdot ({I}_{{L}_{1},act}+{I}_{{L}_{2},act}+{I}_{{L}_{3},act})\cdot \frac{1}{1000}$
reactive_power_kVar	kVar	float	Calculated based on the voltage *U* of one phase and the respective reactive currents with $U\cdot ({I}_{{L}_{1},rea}+{I}_{{L}_{2},rea}+{I}_{{L}_{3},rea})\cdot \frac{1}{1000}$
apparent_power_kVA	kVA	float	Calculated based on the voltage *U* of one phase and the respective apparent currents with $U\cdot ({I}_{{L}_{1},app}+{I}_{{L}_{2},app}+{I}_{{L}_{3},app})\cdot \frac{1}{1000}$
l1_active_current_A (abbr. ${I}_{{L}_{1},act}$)	A	float	Active current of *L*_1_ computed on gateway based on (RMS) current measured at sensor and the phase shift with respect to the voltage *U* of one phase measured at gateway
l1_reactive_current_A (abbr. ${I}_{{L}_{1},rea}$)	A	float	Reactive current of *L*_1_ computed on gateway based on (RMS) current measured at sensor and the phase shift with respect to the voltage *U* of one phase measured at gateway
l1_apparent_current_A (abbr. ${I}_{{L}_{1},app}$)	A	float	Apparent RMS current of phase *L*_1_ measured at the sensor
l1_power_factor_cos_phi	—	float	Power factor of phase *L*_1_ based on phase shift between current and voltage
l2_active_current_A (abbr. ${I}_{{L}_{2},act}$)	A	float	Analog to phase *L*_1_
l2_reactive_current_A (abbr. ${I}_{{L}_{2},rea}$)	A	float	Analog to phase *L*_1_
l2_apparent_current_A (abbr. ${I}_{{L}_{2},app}$)	A	float	Analog to phase *L*_1_
l2_power_factor_cos_phi	—	float	Analog to phase *L*_1_
l3_active_current_A (abbr. ${I}_{{L}_{3},act}$)	A	float	Analog to phase *L*_1_
l3_reactive_current_A (abbr. ${I}_{{L}_{3},rea}$)	A	float	Analog to phase *L*_1_
l3_apparent_current_A (abbr. ${I}_{{L}_{3},app}$)	A	float	Analog to phase *L*_1_
l3_power_factor_cos_phi	—	float	Analog to phase *L*_1_
lpen_apparent_current_A	A	float	Apparent RMS current of PEN conductor measured at the sensor

**Table 4:** Description for columns of the data record feeder metadata. A row specifies the LV feeder metadata from the date until the next chronological row for that feeder. Each row is uniquely identified by the feeder and the date. The data comprises the number of housing units as well as industry and commerce, installed power of equipment and aggregated industrial consumption data.

Column name	Unit	Data type	Description
feeder	count	int	Pseudonymization of the feeder
substation	count	int	Pseudonymization of the substation
date	—	string	Metadata in the row is valid from this date until the next row of the LV feeder
housing_units_count	count	int	Number of housing units (houses or apartments)
industry_and_commerce_count	count	int	Number of industry and commerce
storage_heaters_kW	kW	float	Installed power of storage heaters
heat_pumps_kW	kW	float	Installed power of heat pumps
electric_heaters_kW	kW	float	Installed power of electric heaters
hot_water_tanks_kW	kW	float	Installed power of hot water tanks
flow-type_heaters_kW	kW	float	Installed power of flow-type heaters
EV_chargers_kW	kW	float	Installed power of EV chargers
public_lighting_kW	kW	float	Installed power of public lighting
other_consumers_kW	kW	float	Installed power of other consumers, e.g. inductive loads
batteries_kW	kW	float	Installed power of batteries
PV_systems_kW	kW	float	Installed power of PV systems
chp_kW	kW	float	Installed power of CHPs
g0_general_kWh	kWh	float	Hourly average consumption of commerce and industry over a longer period of time from type general
g1_workdays_kWh	kWh	float	Hourly average consumption of commerce and industry over a longer period of time from type workdays
g2_evening_kWh	kWh	float	Hourly average consumption of commerce and industry over a longer period of time from type evening
g3_continuous_kWh	kWh	float	Hourly average consumption of commerce and industry over a longer period of time from type continuous
g4_shop_hairdresser_kWh	kWh	float	Hourly average consumption of commerce and industry over a longer period of time from type shop/hairdresser
g5_bakery_kWh	kWh	float	Hourly average consumption of commerce and industry over a longer period of time from type bakery
g6_weekend_kWh	kWh	float	Hourly average consumption of commerce and industry over a longer period of time from type weekend

**Table 5:** Description for columns of the data record weather data. The data is provided at a resolution of one hour, and each row is uniquely identified by the feeder and the timestamp (UTC). The data covers solar radiation, temperature, precipitation, snow, humidity and wind.

Column name	Unit	Data type	Processing	Description
feeder	count	int	—	Pseudonymization of the feeder
timestamp_UTC	—	string	—	Timestamp in time zone UTC
timestamp_CET_CEST	—	string	—	Timestamp in local time zone of the DSO (Central European (Summer) Time)
direct_radiation_W/m2	$\frac{W}{{m}^{2}}$	float	average	Hourly average of the downward solar direct radiation flux at the surface
diffuse_radiation_W/m2	$\frac{W}{{m}^{2}}$	float	average	Hourly average of the downward solar diffuse radiation flux at the surface
air_temperature_C	°C	float	instantaneous	Temperature at 2 meters above ground
precipitation_kg/m2	$\frac{kg}{{m}^{2}}$	float	accumulated	Hourly total precipitation such as rain or snow
snow_height_m	*m*	float	instantaneous	Snow depth
humidity_kg/kg	$\frac{kg}{kg}$	float	instantaneous	surface specific humidity
max_wind_gust_m/s	$\frac{m}{s}$	float	maximum	Maximum wind gust at 10m above ground, collected over hourly intervals
meridional_wind_m/s	$\frac{m}{s}$	float	instantaneous	Meridional wind at 10m above ground
zonal_wind_m/s	$\frac{m}{s}$	float	instantaneous	Zonal wind at 10m above ground

Each detailed table includes for each column of the data record the name, unit, data type, and a brief description. All three data records include a *feeder* column, which is an integer specifying the feeder (ranging from 1 to 200). Additionally, the feeder measurements and the weather data include *timestamp* columns in both UTC and CET/CEST which is the local time of the substations. The metadata only includes a date. The unique identifier (primary key) for each data point in the datasets is a combination of the feeder plus timestamp (UTC) or date. Part of the 200 LV feeders selected in the data selection process share the same substation which can be identified by the same voltage values. The metadata includes a column *substation* showing which LV feeders belong to the same substation.

Additionally, we provide a code companion available on GitHub^30^. It comprises functions for downloading the data from Zenodo, loading and joining the data as well as minimal examples for load forecasting and pseudo-measurement generation.

### Feeder measurement data

The feeder measurement data comprises 209,189,649 rows and 20 columns, with the structure detailed in Table 3. For each minute and LV feeder, the dataset includes the voltage of one phase, power values for the sum of all three phases, current values for each phase (including PEN), and power factors for each phase, unless data is missing for the specific feeder and timestamp. PEN stands for protective earth neutral and describes a conductor which is used for grounding and as the neutral conductor. A timestamp, for example April 1, 2023 at 10:05 am specifies that the measurement was taken a few milliseconds before 10:05 am at that day. We choose to represent the data in the units V, A, kW, kVAr and kVA which are most suited for the voltage, current and power ranges in LV grids. Consequently, with the exception of the power values, only the first three decimal places are relevant. The additional decimal places are present due to the floating point representation.

Positive active power indicates a power flow from the MV grid to the LV grid whereas the active power is negative if the power flows from the LV grid in the direction of the MV grid. The reactive power is positive for inductive behavior and negative for capacitive behavior. Additionally, if the voltage is zero, a default value of 230 V is used to facilitate the calculation of apparent, active, and reactive power values. The data is stored in parquet format to enable efficient compression of the extensive dataset^31^. The parquet format is column-oriented which means that the data is stored by column and not by row.

### Feeder metadata

Table 4 outlines the columns of the feeder metadata, where each of the 23 columns (except *feeder*, *substation* and *date*) represent metadata attributes of the feeder, 20 in total. Due to the relative stability of feeder metadata compared to dynamic feeder measurements and weather data, the data record contains only 1,608 rows. Each row specifies the metadata of a LV feeder for the time interval starting at the row’s date and ending at the date of the next chronological row for that feeder.

The feeder metadata includes the number of housing units and industry and commerce in the underlying LV grid of the feeder. Furthermore, it includes the installed power of consumers such as various heating systems, EV chargers, public lighting and other consumers. The installed power of batteries can be both load and feed-in. Feed-in is provided by PV and combined heat and power (CHP) systems.

To enhance information regarding industry and commerce, the dataset includes aggregated average hourly energy consumption data for seven different customer types. It is important to clarify that these values represent averages over long periods (months or years) rather than a time series of hourly consumption data. The values are updated irregularly because the consumption data originates from several industrial and commercial customers with different measurement settings. The six different customer groups of industry and commerce plus the general category *g*_0_ follow the classification proposed in^32^ for representative load profiles. However, this only applies to the classification of customer groups, the data itself has nothing to do with representative load profiles. Customer type *g*_1_ is for load during workdays from 8 a.m. to 6 p.m. while *g*_2_ is for industrial/commercial customers which predominantly consume in the evening hours. Customer type *g*_3_ is from buildings with a constant load demand. Shops are categorized into *g*_4_ while the bakeries get an extra category with *g*_5_. The category *g*_6_ is industry and commerce with its main consumption on the weekend. Finally, *g*_0_ is for all industry and commerce not falling into *g*_1_ - *g*_6_.

The dataset is stored in CSV format to ensure compatibility with programs that may not support compressed parquet files, facilitating quick and easy access to the data.

### Weather data

The weather data described in Table 5 corresponds to individual feeders and their respective timestamps. The weather data for feeder *i* is specific to the postal code where the LV feeder *i* is located. The dataset comprises 3,508,800 rows and 12 columns, including direct and diffuse radiation, air temperature, precipitation, snow height, humidity, maximum wind gust, meridional wind, and zonal wind. Global radiation is the sum of direct and diffuse radiation. Meridional wind is the wind speed in north-to-south or south-to-north direction and zonal wind represents the wind speed in east-to-west or west-to-east direction. The column *Processing* describes if the value is an average for this hour, for one point in time (instantaneous) within this hour, accumulated throughout the hour or the maximum of the hour. For example, *direct_radiation_W/m2* with timestamp April 1, 2023 at 10 am is the average direct radiation between 10 and 11 am.

## Technical Validation

In this section we provide more information about why the data is plausible. We focus on the feeder measurements and the feeder metadata. In particular, we analyze active power and related feeder metadata. Active power is often used in applications such as LV load forecasting and it is based on several measurements recorded in Table 3.

However, we also implemented checks for the other measurement values which are the basis for the sections about limitations of feeder measurement data, feeder metadata and weather data. It comprises systematic checks for each data point such as checking for each phase if (1) the square of the apparent current equals the sum of the squares of active and reactive current, (2) the apparent power is always greater than or equal to active/reactive power, (3) the power factor equals active divided by the apparent current, (4) the signs of the power factor and the active current are the same and (5) that there is no consumption (g0-g6) if no industry and commerce is connected to the LV feeder. Furthermore, individual sanity checks are used including (1) if the feed-in and solar radiation resemble the expected feed-in and solar radiation for timezone CET/CEST, (2) the direct and diffuse radiation between 10 p.m. and 4 a.m. is below 1 $\frac{W}{{m}^{2}}$ and (3) there is no snow between May and September. In addition, we checked for all columns in the feeder measurement data and weather data how often measurement values are repeating for consecutive timestamps. For example, it is plausible that direct/diffuse radiation repeats the same value of 0 $\frac{W}{{m}^{2}}$ during the night but it is unrealistic that the value remains constant throughout the day.

### Typical patterns in weekly profiles

**Fig. 4:**
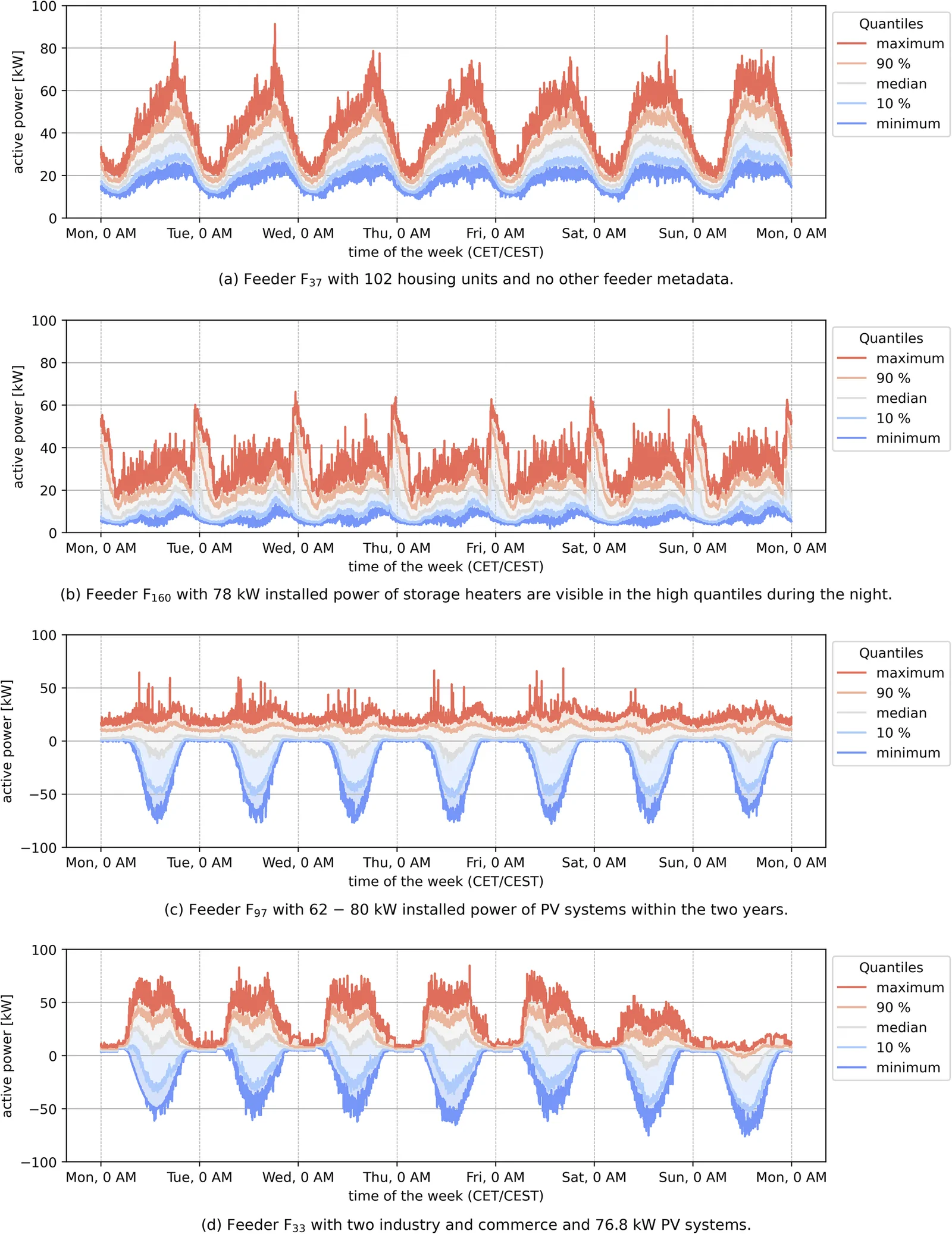
Active power weekly quantile profiles from four LV feeders showing diverse characteristics. Constructed by shrinking the two-years measurements to one week and taking the quantiles for each minute of the week. The metadata of the LV feeders is in Table 6.

Figure 4 presents aggregated weekly quantile profiles for four different LV feeders of active power. This involves condensing the two-year active power time series for each feeder into a single week, assigning approximately 104 to 105 data points to each minute of the week, and then calculating the quantiles. The periodic nature facilitates visual validation through aggregated weekly data. The LV feeders exemplify four distinct patterns, evident in their power profile due to unique periodicity. In Table 6 we provide the corresponding metadata information. The metadata itself already indicates that the LV feeders are characterized by housing units (*F*_37_), storage heaters (*F*_160_), PV systems (*F*_97_) and industry and commerce (*F*_33_).

**Table 6:** Minimum and Maximum of the feeder metadata of the six LV feeders which are visualized in the *Technical Validation*.

LV feeder^a^	hous. (count)	indus. (count)	sto. h. (kW)	heat p. (kW)	e. hea. (kW)	hot w. (kW)	EV (kW)	batt. (kW)	PV (kW)	g (kWh)
*F*_37_	102	0	0	0	0	0	0	0	0	0
*F*_160_	47–48	10	78	0	0	15	0	5–9	6–14	4
*F*_97_	7–12	0	2–11	11–25	8	9–18	33–66	18–28	62–80	0
*F*_33_	0	2	0	0	0	0	0–44	0	77	8–15
*F*_132_	47	0	15	12	0	11	11–22	0–16	79–103	0
*F*_63_	13–15	5–6	68	0	11	0	11	0–11	6–78	0

For every LV feeder, the number of units, the installed power and the average consumption by industry and commerce is given. Values are rounded to the nearest whole number. Consumption of industry/commerce is summarized in one column *g*. Metadata which is zero for all six LV feeders is not shown. ^a^abbreviations of column names: hous. (housing_units), indus. (industry_and_commerce), sto. h. (storage_heaters), heat p. (heat_pumps), e. hea. (electric_heaters), hot w. (hot_water_tanks), EV (EV_chargers), batt. (batteries), PV (PV_systems).

Figure 4a illustrates the LV feeder *F*_37_, characterized only by 102 housing units. The load increases from morning to evening and decreases overnight, with a notable baseload as the lowest values remain significantly above 0 kW. Additionally, the load at the weekend is higher in the late morning and at noon compared to weekdays, possibly due to more people being at home.

Figure 4b depicts the weekly active power profile of LV feeder *F*_160_, which connects, among other things, 47–48 housing units, 10 industry and commerce units, and 78 kW of storage heaters to the grid. The impact of these storage heaters is evident near midnight, with sharp increases and decreases in load in the median and higher quantiles. This is expected, because storage heaters are activated synchronously.

In contrast to *F*_37_ and *F*_160_, the LV feeder *F*_97_ in Fig. 4c includes a significant installed power of PV systems with 62–80 kW over the two years. Accordingly, we observe feed-in from the LV feeder to the MV grid, as indicated by the minimum crossing  −50 kW.

Figure 4d presents feeder *F*_33_ with two units of the category industry and commerce and no housing units at all. Increased load during typical working hours and a reduced demand for load during the weekend can be observed. Furthermore, feed-in from the PV systems with 77 kW is visible in the low quantiles, with even lower values during the weekend.

We do not go into detail regarding quantile plots for other columns in Table 3. Nevertheless, we note that periodic behavior is also observed in several other columns, such as reactive power with periodicity linked to industrial activity or PV system feed-in. Since both motors and pumps in industry or inverters of PV systems have an influence on reactive power, this is expected.

### Seasonal effects on power values

**Fig. 5:**
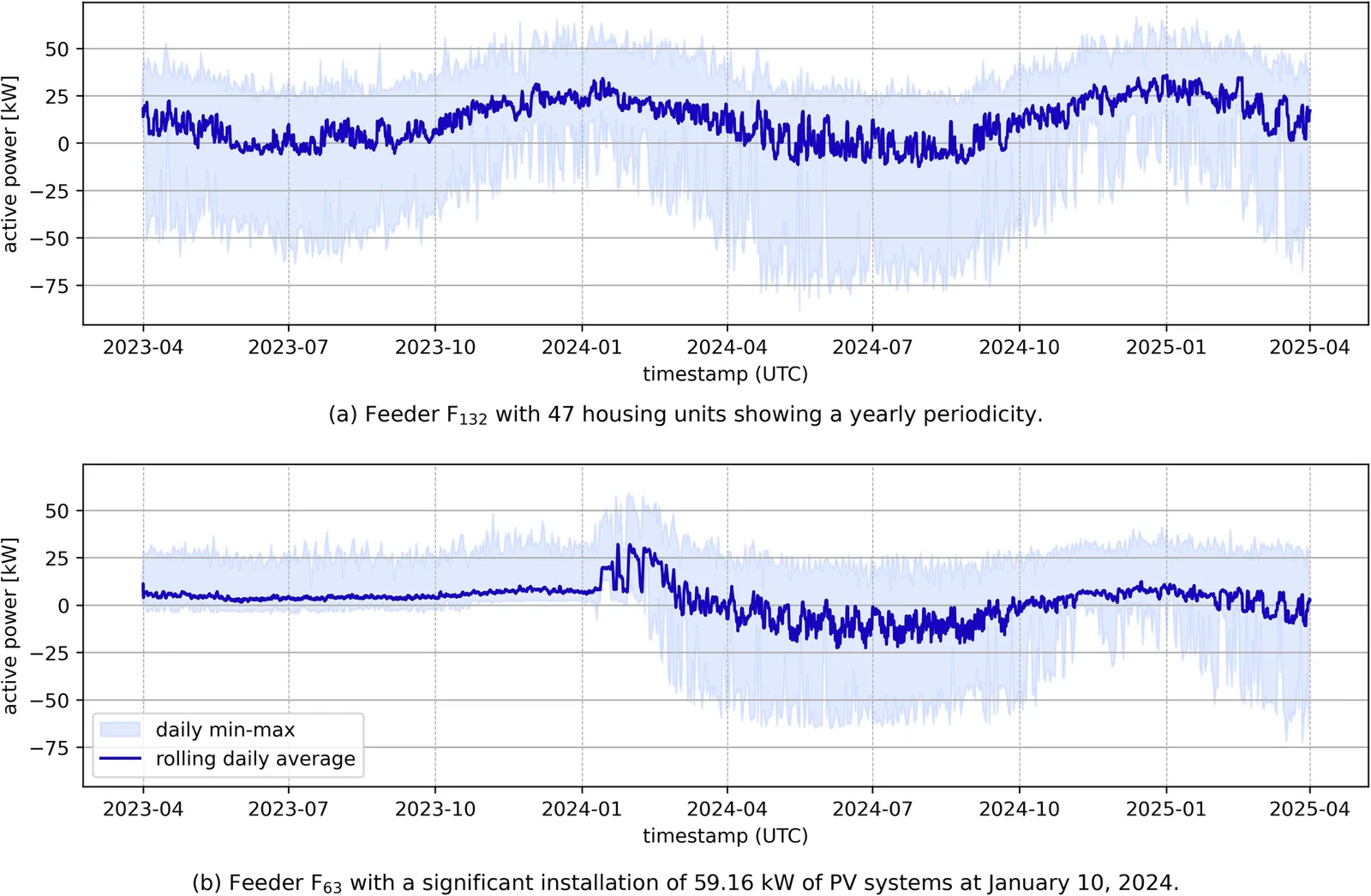
Active power plotted over two years for selected LV feeders. A seasonal effect in winter and summer is visible through higher loads and higher feed-in.

Figure 5 illustrates the rolling daily average together with the daily minimum and maximum values for the two-year active power values of two LV feeders. *F*_132_ in Fig. 5a shows a yearly periodicity with increased feed-in during summer and higher load in winter. An increase of the maximum feed-in in the second year compared to the first year is visible for *F*_132_. Figure 5b highlights a concept drift in the active power of feeder *F*_63_ over two years, attributable to the installation of PV systems on January 10, 2024, which significantly increased feed-in. This drift allows for a comparative analysis of data before and after the PV installation. The feeder *F*_63_ also exhibits a concept drift with respect to load in January and February 2024 which cannot be explained by the feeder metadata. A possible explanation is untracked topology changes.

### Effect of power flow reversal

We like to point out that the equation $S=\sqrt{{P}^{2}+{Q}^{2}}$ for apparent power *S*, active power *P* and reactive power *Q* is not applicable at the LV feeder level combining all three phases. A major reason are valid measurements representing opposite current and power flows on the phases. When calculating $x=| S-\sqrt{{P}^{2}+{Q}^{2}}|$ for all data points, then *x* = 38.06 kVA for LV feeder *F*_127_ on April 9, 2023 13:49 CEST is the largest value. For this timestamp, the power factors *λ* are ${\lambda }_{{L}_{1}}=-1$, ${\lambda }_{{L}_{2}}=-1$ and ${\lambda }_{{L}_{3}}=1$, indicating opposite power flows of the phases. However, if the data points are filtered based on *y* < −2 or *y* > 2 with $y=\sum {\lambda }_{{L}_{1}}+{\lambda }_{{L}_{2}}+{\lambda }_{{L}_{3}}$, *x* = 9.46 kVA is the largest value for *F*_9_ on November 3, 2023 10:27 CET with *y* = 2.09.

Opposite flows on the phases can be caused by single-phase inverters of PV systems. The choice between using active or apparent power in experiments depends on the research question. The active power indicates a net load or net feed-in of the underlying LV grid. In contrast to the apparent power, the active power also includes a direction of the power flow. However, during times of high reactive power flows or opposite power flows on the phases, the active power is insufficient to indicate the thermal utilization of the cable or overhead line. The example of LV feeder *F*_127_ on April 9, 2023 13:49 CEST illustrates this. Here, the active currents ${I}_{{L}_{1},act}=-43$ A, ${I}_{{L}_{2},act}=-48$ A and ${I}_{{L}_{1},act}=84$ A almost cancel each other out and the reactive currents are between  −2 A and 1 A. This results in a low active power of  −1.7 kW and a low reactive power of  −0.5 kVar while at the same time the apparent power is 39.9 kVA. Hence, the active power at the LV feeder level for all phases is not reflecting the thermal load in this case.

Regarding the power factor, we observe that the values change quickly within a few minutes if the direction of the power flow changes. This is often the case shortly after sunrise and shortly before sunset when the PV feed-in increases or levels off. The direction flips the power factor to the opposite, because the power factor *λ* is defined by $\lambda =\frac{P}{S}$. Furthermore, the current is very low and therefore out of the effective range of the sensor during the transition of the power flow direction. Consequently, the inversion of the power flow direction and the decreased accuracy due to low currents lead to fluctuations in the power factor.

### Two-years increase of equipment installations

**Fig. 6:**
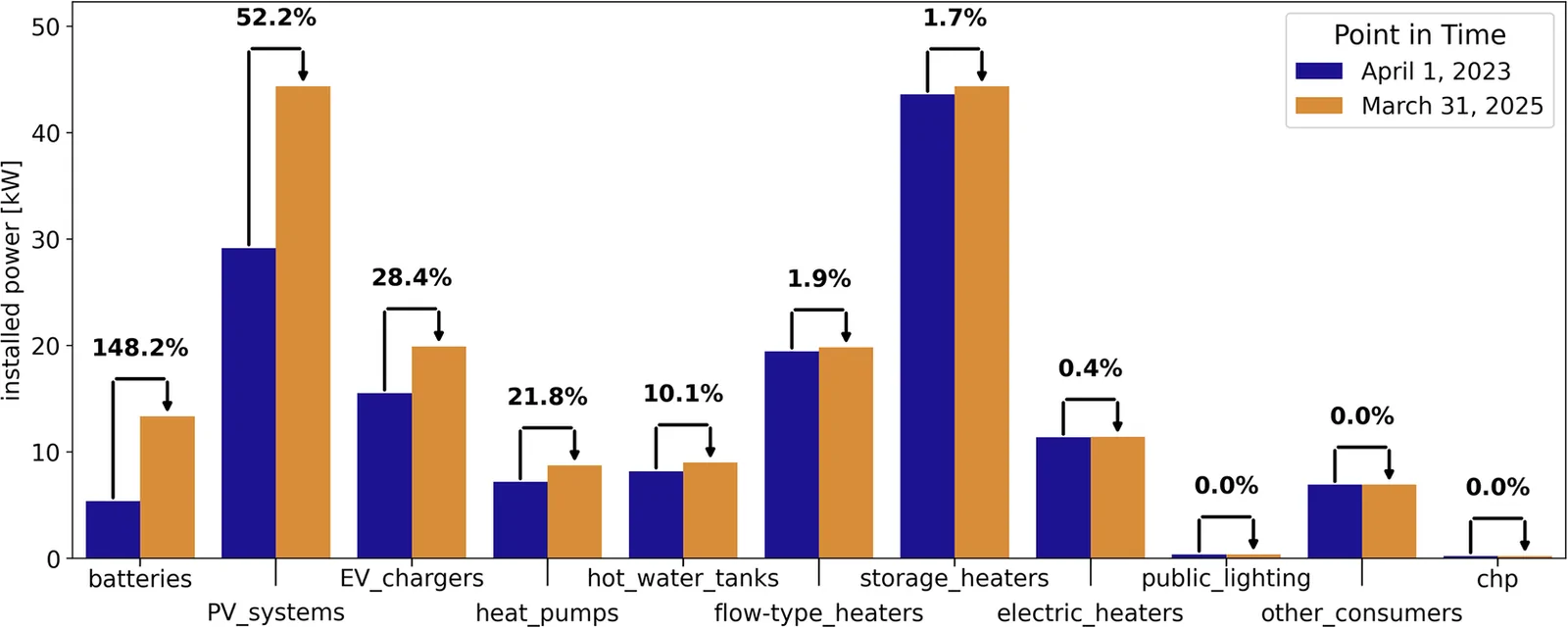
Average installed power per LV feeder of all consumers and producers in the feeder metadata from April 1, 2023 to March 31, 2025. The increase of typical LCTs characterizing the energy transition is clearly visible. The ‘_kw’ suffix of each column name was removed.

In Fig. 6, we observe the change in the mean installed power per LV feeder for consumers and producers from the first day of measurement data in 2023 to the last day in 2025. A clear increase in installed power is evident for specific equipment, notably batteries, PV systems, EV chargers, and heat pumps, which are key drivers of the energy transition. Batteries exhibit the highest increase of 148.2%, driven by widespread installations in Germany due to falling prices and government support programs^33^. The installed power of PV systems rises by 52.5% across the 200 feeders, starting from an average of 29.08 kW per LV feeder^34^. EV chargers and heat pumps also show significant increases of 28.4% and 21.8%, respectively, though these were less pronounced compared to batteries and PV systems, aligning with lower-than-expected sales of new EVs and heat pumps^35^. For other metadata only hot water tanks exhibited a notable increase of 10.1%, with all other changes remaining below 2%.

### Limitations in the feeder measurement data

Even though we only visualize active power values in the technical validation (see Fig. 4 and 5), the feeder measurement data comprises many more electrotechnical variables as depicted in Table 3. As already mentioned in the beginning of this section, we conducted extensive checks and validation to all of them to ensure a good quality of data and be able to describe limitations.

#### Missing measurements

The dataset comprises one-minute LV feeder measurements from 200 LV feeders, with an expected total of 210,528,000 measurements. The expected number of rows results from 200 LV feeders measured over two years whereas one year has 365.5 days, 24 hours and 60 minutes. The feeder measurement data record contains 209,189,649 rows, indicating a completeness of 99.36% and only 0.64% missing values. Regarding missing values in existing rows (NULL values), there are only 833 measurements of voltage missing (0.0004%) and 25.6% of PEN values. As described in the methods, 51 LV feeders have no PEN values and *F*_181_ has only around two third because they were removed in a postprocessing step. In most cases, the reason is that the instrument transformer could not be installed to measure the PEN due to limited installation space in the substation.

#### Outliers in measurements

For some LV feeders, outliers are present in the measurement data, such as unusually high active or reactive power values resulting from high current measurements. The causes of these outliers are diverse and often difficult to identify or correct, as they may reflect valid measurements. Therefore, we did not do any postprocessing regarding outliers. Only one current value is greater than the effective measurement range of 400 A (*F*_134_ on August 12, 2023 15:01 CEST). The same applies to the upper bound of the voltage, which has one value exceeding the effective measurement range of 264 V (*F*_16_ and *F*_88_ from the same substation on July 5, 2023 10:24 CEST). Regarding the lower bound of the voltage measurement range, 71 data points are below 85 V. Apart from two consecutive data points of *F*_145_, all data points belong to *F*_38_, *F*_94_ and *F*_187_. These three LV feeders show a significant voltage drop for 77 minutes in the period November 4, 2024 11:47 CET to November 4, 2024 13:04 CET. It is unclear if this is a valid measurement.

#### Voltage measurements

The user manual does not specify a full scale error for the voltage^24^. Hence, the voltage accuracy is fully given with 3% between 85–264 V for the measured phase. The voltage is measured only on one phase and the voltage of the measured phase is assumed for the other phases. This can lead to inaccuracies for the calculation of power quantities and phase angles when the magnitude or phase of the voltage differs between the phases (voltage unbalance). On the one hand, the voltage is part of the power equations in Table 3. On the other hand, the phase shift between voltage and current is determined on the gateway to compute power factors as well as active and reactive currents.

An important cause of voltage unbalance is single-phase or double-phase loads and generation, since current unbalance causes voltage unbalance due to the line impedance^36^. Hence, the measured current unbalance may also indicate voltage unbalance. However, voltage unbalance cannot be determined and is not the focus of this dataset. The European norm EN 50160 regulates that under normal conditions, the voltage unbalance should stay under 2% for 95% of the ten minute mean RMS values during each period of one week. However, we would like to point out that some studies examining the effect of single-phase PV systems^37^ or single-phase EV charging^38^ show that voltage unbalance may exceed this limit. For some of the PV systems connected to the LV feeders in the FeederBW dataset, we have strong evidence that they are only connected to one phase due to existing power flows in reverse directions for different phases.

Despite inaccuracies in the face of voltage unbalance, it should be noted that the measurement methodology does not cover harmonic analysis. Hence, the total harmonic distortion (THD) of the voltage is neither measured nor reported in the data. Distortions are deviations from perfect sinusoidal waves due to higher frequency components, for example at 150 Hz (3rd order). When sampling the voltage in the gateway, the harmonics present in the voltage sine wave are not filtered out. This causes inaccuracies in the calculations where the voltage is used, for example with respect to the active and reactive power, which are slightly distorted by a portion of the deformed power. The measurement methodology does not allow specifying the deformed power separately.

#### Current measurements

In contrast to the voltage measurements, the effective current is measured for each phase. Hence, the current unbalance can be determined and the accuracy for the apparent currents of each phase is given with 3% between 5–400 A^24^. Regarding the breakdown into active and reactive currents, we already noted that the single-phase measured voltage affects the active and reactive currents. The reason is that they depend on the phase shift which is determined between the phase of the respective current and the single-phase measured voltage.

Just like voltage, current can also deviate from the ideal sine wave. This current distortion is not measured or explicitly stated in the data records. Analogous to the voltage, sampling the current waveform incorporates harmonic influence which is not filtered out. Loads and generation introducing current distortion comprise non-linear loads such as electronic appliances^39^. However, neither current nor voltage distortion is the focus of this dataset. Harmonic analysis requires more sophisticated measurement methodology, for example a power quality analyzer as used in^39^ to determine the unbalance of current harmonics.

#### Low current levels

We note that low currents may not be recorded due to the energy harvesting requirement of the sensors around each phase. The total current, i.e. the sum over all phase currents and the PEN, must exceed 32 A for a continuous measurement according to the user manual^24^. Hence, measurements of a theoretical maximum of 32 A on one phase can be missing if the total current was too low. However, when only taking into account rows where all phase currents and the PEN are existing, the total sum of the currents is below 32 A for 6.13% of the rows. This indicates that the sensor also captures many values when the total current is below the requirement of 32 A. However, this is not guaranteed. Measurements taken at timestamps with a total current of less than 32 A are not subject to increased inaccuracy due to the energy harvesting. Nevertheless, low current values exhibit a worse signal-to-noise ratio compared to higher current values, which increases the error. A full scale error for low values is not specified in the user manual. Hence, the measurement accuracy is only given as 3% for currents between 5 −400 A^24^.

### Limitations in the feeder metadata

The feeder metadata can be considered as time-series event data and therefore we do not have missing timestamps analog to the feeder measurements. We carefully conduct the assignment of metadata to a LV feeder. However, we point out that the assignment can be inaccurate for some LV feeders due to technical restrictions. Furthermore, we note that equipment in the LV grid can be unregistered. This implicates that existing feeder metadata can be missing or wrong in the data record. Furthermore, the day of registration in the feeder metadata and the day for the start of operation can differ, for example when PV systems are registered but not yet active.

The assignment of feeder metadata is only at the level of LV feeders and there is no assignment of feeder metadata to the individual phases. However, we provide information about typical single-phase and three-phase devices in the usage notes.

During operation over the two-year period, switching events in the grid altered the topology, often due to construction work. These events are not documented in the data and normally lead to concept drifts. While obvious cases were removed, some may still be present. In such instances, the feeder metadata may not accurately represent the consumers and producers, affecting the measurement data at the feeder sensor. In contrast, natural concept drifts, such as changes in weather or customer behavior, also occur. Concept drifts can additionally manifest in reactive power, influencing apparent power.

**Fig. 7:**
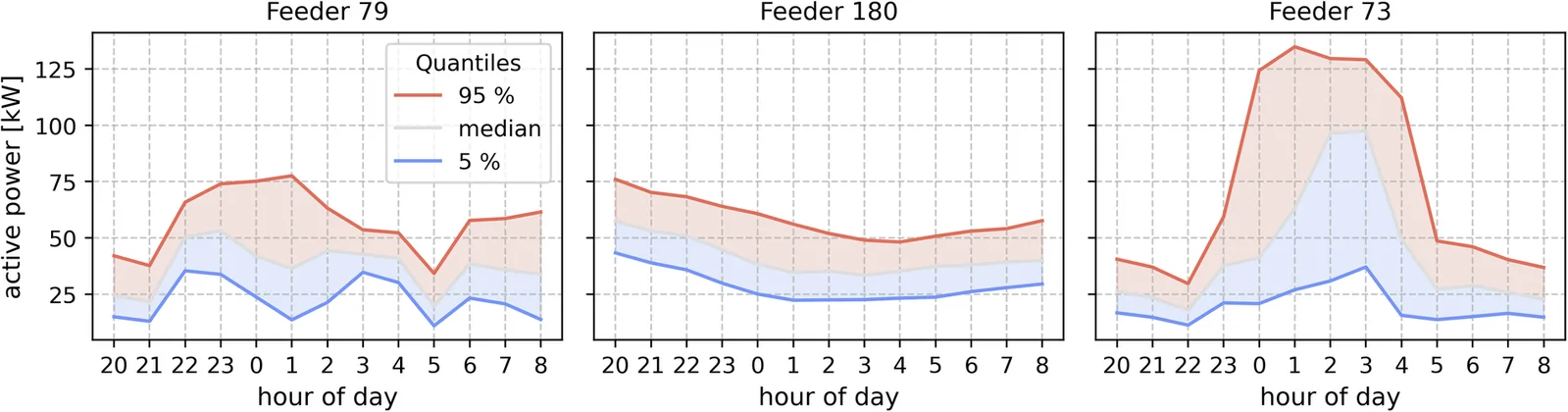
Quantiles of active power during the night times in December, January and February for three LV feeders with high installed power of storage heaters according to the metadata. The values are 211.5 kW (*F*_79_), 181.5 kW (*F*_180_) and 166.5 kW (*F*_73_). The profiles indicate that storage heaters of LV feeders can be active, partly active or not active at all.

Storage heaters, a technology primarily installed decades ago, remain active and are observable in measurements due to their periodic activation during winter nights. Figure 7 shows the quantiles of the active power profiles for three LV feeders with high values of registered storage heaters in the metadata during night times in winter. It can be observed that the profiles differ significantly during times where the demand of storage heaters should be dominating or at least visible. This indicates that storage heaters of LV feeders can be active, partly active or not active at all. Hence, the metadata may not accurately reflect the power of these storage heaters if they are not deregistered with the DSO. Automatic removal of incorrect storage heater data is challenging due to varying activation times and usage intensity. Furthermore, missing information about already deregistered equipment affects also the other consumers and producers.

**Fig. 8:**
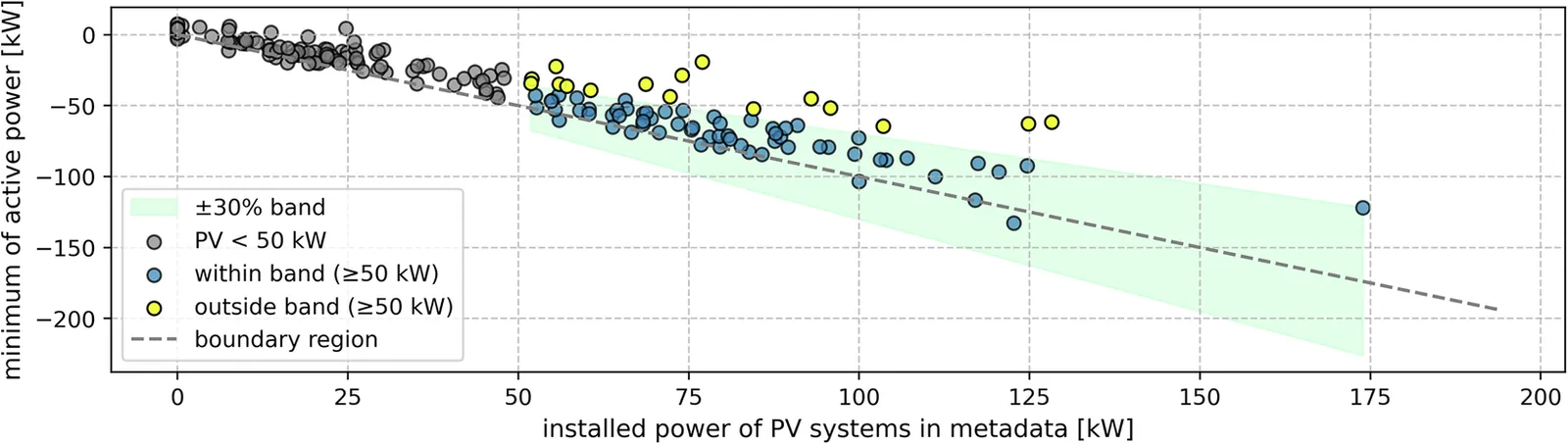
Comparison of the installed power of PV systems in the metadata with the observed minimum of active power. We conduct a plausibility check by counting how many feeders with registered PV capacity above 50 kW exhibit a corresponding minimum in the active power measurements within a 30% band. The figure shows that 113 (58.9%) are ignored due to the 50 kW threshold (gray), 63 (32.8%) are within the band (blue) and 16 (8.3%) are outside the band (yellow). In total, 7.3% of the 192 feeders shown in the figure exhibit a lower minimum in active power compared to the installed PV capacity in the metadata. Eight LV feeders with additional generation in the form of CHPs registered are excluded from the figure.

Figure 8 contrasts the installed power of PV capacity according to the metadata with the observed minimum of active power for LV feeders where only PV systems feed into the grid. There is a clear correlation visible with a correlation coefficient of  −0.948. Only 7.3% of the LV feeders exhibit slightly lower minimum values of active power compared to the installed power of PVs. We conduct another plausibility check for LV feeders exceeding 50 kW of registered PV capacity. It is observable that 63 LV feeders (32.8%) have their minimum within a band of 30% around the registered PV capacity. In contrast, 16 feeders (8.3%) exhibit minima in active power lower than the registered PV capacity. 113 feeders are ignored for the plausibility check because they are below 50 kW. The reason is that the ratio between load and generation is higher, which leads to a higher influence of load occurring at the same time as the PV generation. Even though the metadata seems plausible with respect to the active power values, we can only validate the metadata to a limited extent. We cannot clearly estimate if the registered PV capacity is too high or too low in the metadata because the production depends on the age, angle, tilt, and degree of soiling of the PVs modules. Furthermore, the generation overlaps with consumption, as it can be observed, for example, for feeder *F*_166_.

Regarding the different customer groups of industry and commerce, it should be noted that even though the distinction in the dataset is sharp, this is not necessarily the case for individual industrial and commercial customers. Table 7 includes several examples which show that the assignment of customer groups to *g*_0_-*g*_6_ is ambiguous. For example, a bakery is assigned to group bakery (*g*_5_). However, while this is meaningful for a traditional bakery which bakes bread locally and at night, bakeries only selling bread may be more similar to shops and hairdressers (*g*_4_). Another example is restaurants which can have their main electricity usage in the evening (*g*_2_) or on the weekend (*g*_6_). It is possible to sum up the seven columns *g*_0_-*g*_6_ into one column *g*_*sum*_ in order to eliminate the ambiguity.

**Table 7:** Examples for category ambiguity of different customer groups of industry and commerce.

Customer group	g0 (general)	g1 (workdays)	g2 (evening)	g3 (continuous)	g4 (shop)	g5 (bakery)	g6 (weekend)
Bakery					(*✓*)	*✓*	
Restaurant			*✓*				(*✓*)
Clubhouse		(*✓*)	(*✓*)				*✓*
Manufacturing industry		*✓*		(*✓*)			
Sports hall		(*✓*)	(*✓*)				*✓*
Unknown	*✓*	(*✓*)	(*✓*)	(*✓*)	(*✓*)	(*✓*)	(*✓*)

The symbol *✓* indicates that this category is used for the dataset while (*✓*) marks other plausible categories. Users may sum up the columns *g*_0_-*g*_6_ into a single column in order to eliminate multi-label ambiguity.

### Limitations in the weather data

The weather data record does not exhibit missing rows in the dataset, so 100% of the data is existing. Null values only occur in column *snow_height_m*, primarily in the summer and autumn months from June to October in the year 2024. Applications and models can assume 0 m because there is usually no snow at this season.

## Usage Notes

The data can be accessed in Zenodo. The data records have a total size of 8.5 GB (compressed) and are provided in the format parquet and CSV. The feeder metadata and the weather data is provided in one file. The feeder measurement data is contained in 200 files (one file for each LV feeder) and compressed into five zip files with 40 parquet files respectively. The data can be loaded with common python libraries such as polars, pandas or other tools.

We encourage scientists conducting ML experiments with the dataset to follow a consistent split of the data into training and test to enhance comparability. We recognize that deviations may be appropriate depending on the research question or methodology. For locational splits, we recommend to use feeders *F*_1_ − *F*_160_ for training and *F*_161_ − *F*_200_ for testing. When conducting a cross validation, we recommend using a five-fold-cross validation with the folds *F*_1_ − *F*_40_ (test feeders in the first iteration), *F*_41_ − *F*_80_ (test feeders in the second iteration), *F*_81_ − *F*_120_ (test feeders in the third iteration), *F*_121_ − *F*_160_ (test feeders in the fourth iteration) and *F*_161_ − *F*_200_ (test feeders in the fifth iteration). The locational splits correspond to a random distribution.

For temporal splits, we recommend to use the first year (April 1, 2023 to March 31, 2024) for training and the second year (April 1, 2024 to March 31, 2025) for testing. In addition, we encourage users to apply cross validation for time-series forecasting. We do not recommend folds for the time-series cross validation because they can differ based on your research question.

Methods and experiments using the feeder metadata can be designed to be robust against the presented limitations in the feeder metadata. Alternatively, metadata correction techniques can be developed and used before applying other methods, for example based on NILM or various ML-based approaches. Metadata correction techniques may need to consider the complex interactions between load and consumption, weather effects, changes in consumption behavior, seasonal effects or only slightly pronounced concept drifts in LV feeders with high base load. It could be beneficial to not only look at three-phase active power values, but also consider reactive power, power factors and the currents of individual phases.

We shortly summarize some aspects in the context of the dataset that influence the interpretation of the measured data. The capacity of a LV feeder depends on several factors, including the conductor size and the installation. Therefore, it is not possible to derive grid congestions or capacity limits from the data. For example, the ampacity of overhead lines is higher compared to underground cables for the same conductor size^40^. The 200 LV feeders exhibit 12% overhead lines while the remaining are underground cables. Overhead lines are mostly air-insulated.

The standard cable in German LV grids and in the present dataset is an aluminum cable from type NAYY with 150 *mm*^2^^ 41^. The current-carrying capacity (rated current) depends on different factors such as the type of load and installation conditions and is depicted with 270 A per phase in^41^. Other cables contained in the dataset comprise the types NAYCWY with 150 *mm*^2^ and comparable current-carrying capacity around 270 A. Overhead lines are often with lower capacity around 200 A. A LV feeder can consist of different line types and has cable joints connecting grid customers through service lines with significantly lower current-carrying capacity. In the present dataset, the shortest LV feeder is around 100 m and the longest feeder is around 2.1 km (including service lines to customers). Overall, we note that the conductor types and the length of feeders influence impedance, losses and ampacity in the LV grid.

The feeder metadata does not contain information about which devices are connected to which phase of the LV feeder. In Germany and in the present dataset, all grid connection objects, including apartments, are usually connected to all three phases. Furthermore, storage heaters and flow-type heaters are mainly connected to three phases. In addition, recently installed EV chargers, PV systems and CHPs are mostly connected to all three phases. However, we observe in the FeederBW dataset that a significant share of older PV systems is only connected to a single phase. Current unbalance including feed-in is a helpful indicator for the presence of single-phase PV systems at the LV feeder. Single-phase connection may also be present for other devices, for example storage heaters. While hot water tanks can be connected to a single phase or all three phases, heat pumps for housing are typically connected to a single phase. Large heat pumps require a three phase connection. Electric heaters, public lighting and batteries are usually connected to a single-phase. However, from the point of view of the LV feeder, the devices are distributed over different phases.

The data is collected after accounting for grid losses in the LV grid, which are mostly below 1.3% with a median of 0.45% according to comparable literature^42^. Consequently, the influence of network topology and the distance between equipment and measurement at the LV feeder on the measured values is moderate.

Regarding price elasticity and demand response, it is expected to be not or only slightly observable in the measurement data. Even though there is a obligation since 2025 to offer dynamic tariffs to customers and dynamic network charges are present for some customers, a wide usage is not given, in particular due to missing smart meter installations^43^. Some customers are still prized based on a high (often hours during the day) and low tariff (often hours during the night) which was introduced some decades ago to make better use of base load power plants^41^. Some customers may also use special tariffs for large consumers such as heat pumps.

It is important to clarify that the installed power of EV chargers does not directly correspond to the presence of EVs in the grid. Funding programs for private EV charging stations have led to the installation of chargers in anticipation of future EVs.

The dataset does not differentiate between single-family and multi-family houses. However, most LV feeders are located in rural areas, predominantly featuring single-family houses. A higher number of housing units indicates the presence of multi-family houses.

The method of room heating significantly influences the load on the LV feeders. A higher degree of electrification for heating increases the load. During 2023–2025, the majority of houses still rely on fossil fuels such as gas or oil for heating^44^. Gas is commonly used in buildings connected to gas pipelines.

Batteries in the dataset are primarily used to store energy from PV systems and supply it directly to the house. Some batteries also have the capability to draw power from the grid or feed it back, depending on their configuration.

## Data Availability

The data is available under Zenodo (https://zenodo.org/records/19550073).

## Code availability

We provide a code companion available on GitHub^30^. It comprises functions for downloading the data from Zenodo, loading and joining the data as well as minimal examples for load forecasting and pseudo-measurement generation.
